# PPARβ/δ-dependent MSC metabolism determines their immunoregulatory properties

**DOI:** 10.1038/s41598-020-68347-x

**Published:** 2020-07-10

**Authors:** R. A. Contreras-Lopez, R. Elizondo-Vega, M. J. Torres, A. M. Vega-Letter, N. Luque-Campos, M. J. Paredes-Martinez, C. Pradenas, G. Tejedor, K. Oyarce, M. Salgado, C. Jorgensen, M. Khoury, G. Kronke, M. A. Garcia-Robles, C. Altamirano, P. Luz-Crawford, F. Djouad

**Affiliations:** 1 Centro de Investigación Biomédica, Facultad de Medicina, Universidad de Los Andes, Santiago, Chile; 2 Cells for Cells, Consorcio Regenero, Las Condes, Santiago, Chile; 3 Laboratory of Nano-Regenerative Medicine, Facultad de Medicina, Universidad de Los Andes, Santiago, Chile; 4 IRMB, Univ Montpellier, INSERM, CHU Montpellier, Inserm U 1183, IRMB, Hôpital Saint-Eloi, 80 Avenue Augustin Fliche, 34295 Montpellier Cedex 5, France; 5 Facultad de Ciencias Biológicas, Departamento de Biología Celular, Laboratorio de Biología Celular, Universidad de Concepción, Concepción, Chile; 6 Facultad de Ciencias de la Salud, Universidad San Sebastián, Concepción, Chile; 7 Escuela de Ingeniería Bioquímica, Pontificia Universidad Católica de Valparaiso, Valparaiso, Chile; 8 Department of Internal Medicine 3, University of Erlangen-Nuremberg, 91054 Erlangen, Germany

**Keywords:** CD4-positive T cells, Mesenchymal stem cells

## Abstract

Mesenchymal stem cell (MSC)-based therapy is being increasingly considered a powerful opportunity for several disorders based on MSC immunoregulatory properties. Nonetheless, MSC are versatile and plastic cells that require an efficient control of their features and functions for their optimal use in clinic. Recently, we have shown that PPARβ/δ is pivotal for MSC immunoregulatory and therapeutic functions. However, the role of PPARβ/δ on MSC metabolic activity and the relevance of PPARβ/δ metabolic control on MSC immunosuppressive properties have never been addressed. Here, we demonstrate that PPARβ/δ deficiency forces MSC metabolic adaptation increasing their glycolytic activity required for their immunoregulatory functions on Th1 and Th17 cells. Additionally, we show that the inhibition of the mitochondrial production of ATP in MSC expressing PPARβ/δ, promotes their metabolic switch towards aerobic glycolysis to stably enhance their immunosuppressive capacities significantly. Altogether, these data demonstrate that PPARβ/δ governs the immunoregulatory potential of MSC by dictating their metabolic reprogramming and pave the way for enhancing MSC immunoregulatory properties and counteracting their versatility.

## Introduction

Recently, we have identified a novel role of PPARβ/δ in addition to its well described role in lipid catabolism and glucose homeostasis^1^. Indeed, we have demonstrated that the expression level of PPARβ/δ, highly expressed by mesenchymal stem cell (MSC), predicts their immunoregulatory potential and that MSC priming based on PPARβ/δ inhibition enhances their immunoregulatory properties and therapeutic potential in an experimental model of arthritis^2^. However, the role of PPARβ/δ on MSC metabolic activity and the relevance of PPARβ/δ metabolic control on MSC immunosuppressive properties have never been addressed.

PPAR family members are nuclear-receptors that act as transcription factors upon ligand activation and lead to cell transcriptional programming. While PPAR isotypes are found in a large variety of tissues, their expression levels and functions differ according the tissue as revealed by the expression profile of targets. PPARβ/δ expressed at a high level in skeletal muscle is a key regulator of fatty acid oxidation and glucose uptake^3,4^. Thus, in tissues demanding high level of energy, PPARβ/δ increase the expression level of genes associated with fatty acid transport and β-oxidation. In addition, the use of selective PPARβ/δ agonists in vivo has evidenced the anti-inflammatory properties of PPARβ/δ^5^. In macrophages, for instance, the activation of PPARβ/δ induces fatty acid metabolism while represses inflammation^6–8^. In response to cytokines produced by Th2 cell types such as IL13 and IL4, macrophages polarize into alternatively activated macrophages that use oxidative metabolism to fuel their long-term functions^9^ by inducing PPARβ/δ expression in a STAT6 dependent manner^10^. PPARβ/δ invalidation blocks macrophage polarization towards a M2 phenotype^10^. While the role of the PPARβ/δ-dependent metabolic reprogramming in macrophage^9^ and other immune cell phenotype and functions^11^ has been clearly demonstrated its implication remains elusive for MSC immunosuppressive properties.

In the present study, we investigate the role of PPARβ/δ on MSC metabolic activity and the relevance of PPARβ/δ metabolic control on MSC immunoregulatory functions.

## Results

### PPARβ/δ knockdown enhances the capacity of MSC to inhibit the proliferation and the functions of Th1 and Th17 cells

**Figure 1:**
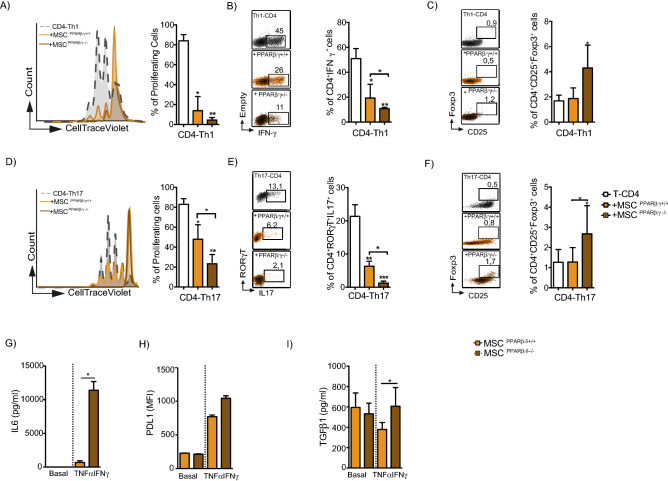
PPARβ/δ silencing increases the immunoregulatory properties of MSC. (**A**–**F**) Naïve T-CD4 murine cells induced to differentiate into Th1 or Th17 cells were labelled with Cell Trace Violet (CTV) and cultured in the presence or absence (white bars) of either PPARβ/δ^+/+^ (yellow bars) MSC or MSC deficient for PPARβ/δ (MSC PPARβ/δ^−/−^) (brown bars). On the left panel, grey histograms represent Th1 (**A**) or Th17 (**D**) cells proliferation alone while yellow and brown histograms represent cells co-cultured with either MSC PPARβ/δ^+/+^ or MSC PPARβ/δ^−/−^ respectively. Representative Dot Plot panels of IFNγ producing-Th1 cells (**B**) or IL17 producing Th17 cells (**E**) cocultured or not with either MSC PPARβ/δ^+/+^ or MSC PPARβ/δ^−/−^. Representative dot plot of Treg generation of Th1 (**C**) or Th17 cells (**F**) cocultured or not with either MSC PPARβ/δ^+/+^ or MSC PPARβ/δ^−/−^. (**G**) IL-6 production, (**H**) PDL1 expression level and (**I**) TGFβ1 production by MSC PPARβ/δ^+/+^ or MSC PPARβ/δ^−/−^ pre-activated or not with pro-inflammatory cytokines. Statistics: non- paired Kruskal–Wallis test. *p < .05; **p < .01; ***p < .001. Unless otherwise indicated, p values refer to values obtained for either CD4-Th1 or CD4-Th17 when cultured alone.

As we previously described^2^, PPARβ/δ inhibition or knockdown in MSC enhances their capacity to inhibit T lymphocyte proliferation. To go a step further, we addressed the effect of PPARβ/δ on MSC immunoregulatory properties focusing on specific T cell subsets including Th1 and Th17 cells. To that end, naïve T-CD4 cells induced to differentiate into both Th1 or Th17 cells were co-cultured with either wild-type MSC (MSC PPARβ/δ^+/+^) or MSC deficient for PPARβ/δ (MSC PPARβ/δ^−/−^). While PPARβ/δ knockout significantly increased the capacity of MSC to inhibit the proliferation of Th1 and Th17 (Fig. 1A,D) and to decrease the percentage of Th1 and Th17 cells (Fig. 1B,E), it also enhanced the capacity to generate regulatory T cells (Treg) (Fig. 1C,F). The immunosuppressive activity of MSC depends on the production of molecules including IL6 and PD-L1. The release of these mediators of MSC immunosuppressive functions is stimulated after 24 h of MSC treatment with TNFα and IFNγ^12^. Therefore, we evaluated the effect of pro-inflammatory cytokines treatment on the capacity of MSC PPARβ/δ^+/+^ and MSC PPARβ/δ^−/−^ to release immunoregulatory mediators and found a significantly higher production of IL6 and TGFβ1 as well as a higher expression level of PD-L1 in MSC PPARβ/δ^−/−^ (Fig. 1G,H,I). These results underline that PPARβ/δ governs the immunosuppressive properties of MSC on Th1 and Th17 cells acting upstream the well-acknowledged MSC immunosuppressive mediators.

### PPARβ/δ knockdown promotes the metabolic switch of MSC toward glycolysis

**Figure 2:**
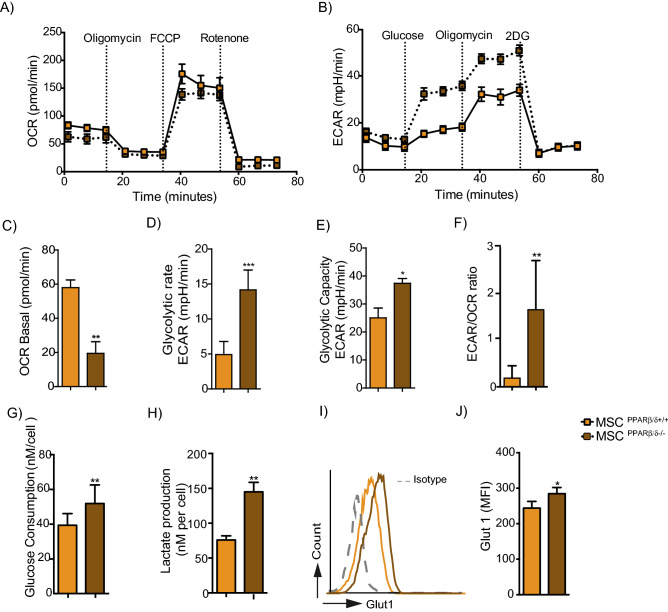
PPARβ/δ silencing induces the metabolic switch of MSC toward glycolysis. (**A**) The metabolic status of MSC PPARβ/δ^+/+^ or MSC PPARβ/δ^−/−^ was determined by analyzing the oxygen consumption rates (OCR) using the Agilent Seahorse XF technology. (**B**) The extracellular acidification rate (ECAR) of the media was also determined using the Agilent Seahorse XF technology. (**C**–**E**) The metabolic status of MSC PPARβ/δ^+/+^ or MSC PPARβ/δ^−/−^ was determined by analyzing the basal oxygen consumption rates (OCR) using the Agilent Seahorse XF technology (**C**). (**D**) The glycolytic rate in terms of extracellular acidification rate (ECAR) quantification of the media and (**F**) the glycolytic capacity were also determined using the Agilent Seahorse XF technology. (**F**) Histogram represents the ratio of ECAR to OCR of either MSC PPARβ/δ^+/+^ (yellow bar) or MSC PPARβ/δ^−/−^ (brown bar). (**G**) Glucose consumption rate (**H**) or lactate production were quantifying from the supernatants of either MSC PPARβ/δ^+/+^ or MSC PPARβ/δ^−/−^ using the YSI equipment. (**I**) Representative histogram of GLUT 1 expression on PPARβ/δ^+/+^ (yellow line) or PPARβ/δ^−/−^ (brown line) MSC and the isotype control (grey dots line). (**J**) Quantification of GLUT1 expression in both PPARβ/δ^+/+^ or PPARβ/δ^−/−^ cells. Statistics: non-paired Mann–Whitney test. *p < .05; **p < .01; ***p < .001. Results are represented as mean ± SD of at least 4 independent experiments.

Since PPARβ/δ is a master regulator of fatty acid oxidation, we wondered whether PPARβ/δ expression could control the bioenergetics profile of MSC. Thus, we assessed in real time the oxygen consumption rate (OCR) to quantify mitochondrial respiration (OXPHOS) (Fig. 2A), and the extracellular acidification rate (ECAR) as an indicator of glycolysis (Fig. 2B). Compared to MSC PPARβ/δ^−/−^, MSC PPARβ/δ^+/+^ exhibited a significantly higher basal respiration rate (Fig. 2C), a lower glycolytic rate and glycolytic capacity (Fig. 2D,E) with no difference in the maximum respiration rate, spare rate capacity (SRC) and glycolytic reserve (Sup. Fig. 1A–C). Regarding the ratio of ECAR to OCR, it was also significantly higher in MSC PPARβ/δ^−/−^ than in MSC PPARβ/δ^+/+^ (Fig. 2F). These results revealed that MSC PPARβ/δ^−/−^ display a higher glycolytic metabolism than MSC PPARβ/δ^+/+^. Then, we evaluated the glucose consumption (Fig. 2G) and lactate production (Fig. 2H) by the MSC and determine their GLUT1 expression profile (Fig. 2I,J). Our results showed that the highly immunosuppressive MSC PPARβ/δ^−/−^ exhibit a significantly higher glucose consumption, lactate production and GLUT1 expression level than their wild-type counterpart suggesting that PPARβ/δ deficiency reprograms MSC metabolism while increases their immunoregulatory functions.

### Oligomycin treatment induces a potent metabolic switch of MSC

**Figure 3:**
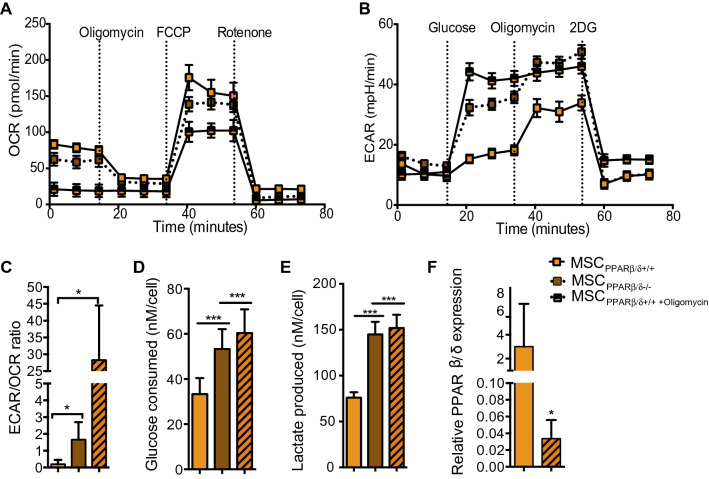
Oligomycin treatment modifies MSC metabolism. (**A**) The metabolic status of MSC PPARβ/δ^−/−^ and MSC PPARβ/δ^+/+^ treated or not with oligomycin was determined by analyzing the oxygen consumption rates (OCR) using the Agilent Seahorse XF technology. (**B**) The extracellular acidification rate (ECAR) of the media was also determined using the Agilent Seahorse XF technology. (**C**) Histogram represents the ratio of ECAR to OCR of either MSC PPARβ/δ^−/−^ or MSC PPARβ/δ^+/+^ treated or not with oligomycin. (**D**) Glucose consumption rate (**E**) or lactate production were quantified from the supernatants of the cells using the YSI equipment. (**F**) Expression level of PPARβ/δ measured by RT-qPCR. Statistics: non-paired Mann–Whitney test. *p < .05; **p < .01. ***p < .001. Results are represented as mean ± SD of at least 4 independent experiments.

Since PPARβ/δ is a master regulator of MSC immunosuppressive property and that the induction of MSC glycolytic metabolism enhanced this latter property, we investigated the effect of a pharmacological metabolic switch on poorly immunoregulatory MSC expressing high level of PPARβ/δ. To that end, we first treated MSC PPARβ/δ^+/+^ for 24 h with oligomycin, an inhibitor of ATP synthase that blocks oxidative phosphorylation, and compared their bioenergetics status to untreated MSC PPARβ/δ^+/+^ and MSC PPARβ/δ^−/−^. MSC PPARβ/δ^+/+^ treated with oligomycin displayed a significantly lower OCR compared to untreated cells (Fig. 3A). Moreover, the treatment of MSC PPARβ/δ^+/+^ with oligomycin significantly increased their glycolytic capacity at a similar level than MSC PPARβ/δ^−/−^ (Fig. 3B). The analysis of the ECAR/OCR ratio of oligomycin treated MSC PPARβ/δ^+/+^ indicated a ~ 30-fold increase compared to untreated PPARβ/δ^+/+^ and a ~ tenfold increase compared to MSC PPARβ/δ^−/−^ (Fig. 3C). Then, we evaluated the glucose consumption (Fig. 3D) and lactate production (Fig. 3E) and showed that oligomycin treatment significantly increased the capacities of MSC PPARβ/δ^+/+^ to consume glucose and produce lactate to the same extent as MSC deficient for PPARβ/δ. To define whether the modulation of MSC metabolism was also associated with PPARβ/δ expression level, we analyzed the expression profile of PPARβ/δ^−/−^ on untreated and oligomycin-treated MSC. PPARβ/δ^+/+^-oligomycin MSC driven toward a glycolytic metabolism exhibited a reduced PPARβ/δ expression level compared to untreated MSC PPARβ/δ^+/+^ (Fig. 3F). These data confirm that the metabolic switch of MSC towards a glycolytic metabolism control PPARβ/δ expression level.

### PPARß/δ-dependent MSC metabolic status partly controls their immunoregulatory potential

**Figure 4:**
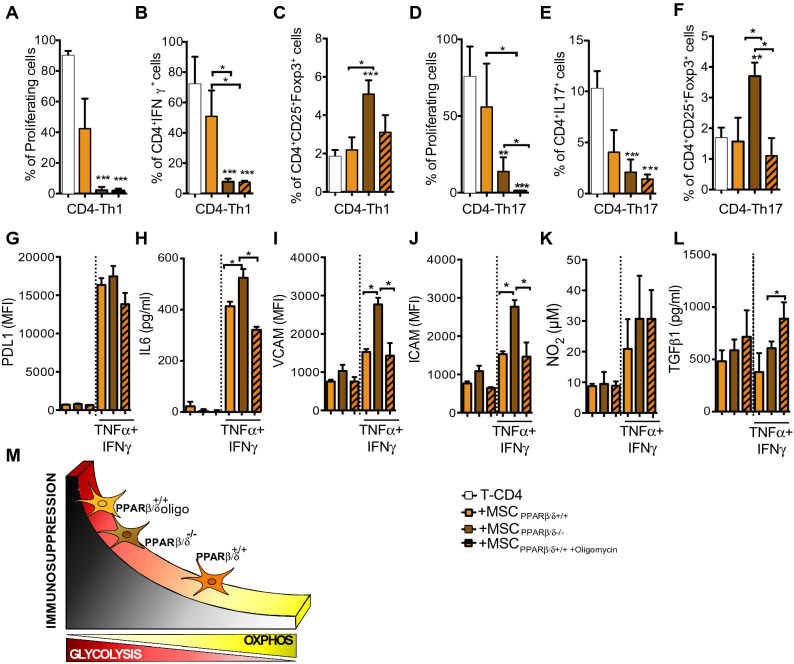
The metabolic status of PPARβ/δ^+/+^ cells drives their immunosuppressive potential. (**A**–**C**) Naïve T CD4 murine cells induced to differentiate into Th1 or (**D**–**F**) Th17 cells were labelled with Cell Trace Violet (CTV) and cultured in the absence (white bars) or presence of either MSC PPARβ/δ^+/+^ pre-treated (lined yellow bars) or not (yellow bars) with oligomycin or MSC PPARβ/δ^−/−^ (brown bars). Proliferation, pro-inflammatory phenotype (IFNγ and IL17 production for Th1 and Th17 respectively) and Treg generation were evaluated by FACS. (**G**–**L**) assessment of PDL1 expression level (MFI, Mean Fluorescence Intensity), IL6 production, VCAM and ICAM expression levels (MFI), NO_2_ and TGF1 production by MSC PPARβ/δ^+/+^ and MSC PPARβ/δ^−/−^ treated or not oligomycin and pre-activated or not with TNFα and IFNβ. Results represent the mean ± SD of 4 independent experiments with 3 different mice each time. Statistics: non-paired Kruskal–Wallis test. *p < .05; **p < .01; ***p < .001. Unless otherwise indicated, p values refer to values obtained for either CD4-Th1 or CD4-Th17 cells when cultured alone. (**H**) Representative figures that shows the association between the metabolic status of MSC with their immunosuppressive potential.

Going further, we decided to study whether the metabolic modulation of MSC PPARβ/δ^+/+^ could enhance their immunosuppressive functions. We performed a co-culture experiments with T-CD4 cells induced to differentiate into Th1 and Th17 cells with either MSC PPARβ/δ^+/+^, oligomycin-treated MSC PPARβ/δ^+/+^ or MSC PPARβ/δ^−/−^. The enhancement of MSC PPARβ/δ^+/+^ glycolytic metabolism significantly increased their immunosuppressive activity on Th1 and Th17 cell proliferation and pro-inflammatory phenotypes (Fig. 4A,B,D,E) to the same level as MSC PPARβ/δ^−/−^ without modifying their capacity to generate Treg cells (Fig. 4C,F). The treatment of MSC PPARβ/δ^−/−^ with a glucose analogue, 2-deoxiglucose (2-DG), that induces OXPHOS metabolism, reduced the capacity of MSC to inhibit the proliferation of T-CD4 cells (Sup. Fig. 2A). Hence, our data demonstrate that the metabolic status of MSC significantly control their immunosuppressive activities partially through a PPARβ/δ-dependent manner. Furthermore, in order to determine whether glucose consumption was responsible for the enhanced immunosuppressive activity of both MSC deficient for PPARβ/δ and MSC pre-treated with oligomycin, we supplemented the culture media with glucose every 24 h. Glucose addition in the media did not modify the proliferation profile suggesting that glucose deprivation is not involved in MSC-mediated immunosuppression (Sup. Fig. 2B). Remarkably, the enhanced immunosuppressive effect of MSC pre-treated with oligomycin was still observed using a transwell system to physically separate activated T-CD4 cells and MSC (Sup. Fig. 2C). Finally, we evaluated the expression profile of some of the mediators associated with the immunosuppressive functions of MSC such as PDL1, IL6, VCAM, ICAM, NO_2_ and TGFβ1. For that purpose, PPARβ/δ^+/+^ and PPARβ/δ^−/−^ MSC were pretreated or not with oligomycin and activated, when indicated, with TNFα and IFNγ. The treatment with oligomycin neither modified the capacity of MSC to express PDL1, VCAM and ICAM nor to produce IL6 (Fig. 4G–J). In contrast, MSC treated with oligomycin produced higher levels of NO_2_ and TGFβ1 as compaerd to the non-treated MSC that reached the levels produced by PPARβ/δ^−/−^ MSC (Fig. 4K,L). Thus, a ranking of MSC immunomodulatory levels according to their metabolic status can be proposed (Fig. 4M).

## Discussion

In the present study, we demonstrated that the enhanced immunoregulatory properties of MSC PPARβ/δ^−/−^ are associated with significantly higher glycolytic capacity, lactate production and higher glucose consumption than wild-type MSC. This result reveals that PPARβ/δ inhibition is a key switch of MSC immunoregulatory functions that acts by promoting MSC metabolic reprogramming towards glycolysis.

Our findings identifies a novel mechanism underlying MSC immunoregulatory properties in which the high glucose consumption by the highly immunosuppressive MSC PPARβ/δ^−/−^ might deprive T cell of glucose and thus impairing their phenotype and functions. Indeed, in lymphoid tissues T cells are primed prior to traffic to sites of inflammation where they will compete for resources with other cell types. Since glucose is pivotal for T cell proliferation and functions, its consumption by MSC and in particular by MSC PPARβ/δ^−/−^ can metabolically restrict T cells, directly altering their function leading to immunosuppression, but this remains to be demonstrated.

While MSC expressing PPARβ/δ did not exhibit any preventive or therapeutic properties in an experimental model of arthritis, MSC deficient for PPARβ/δ exert potent beneficial effects. The inhibition of PPARβ/δ on MSC PPARβ/δ^+/+^ using a selective and irreversible pharmacological inhibitor generates therapeutic cells with both preventive and curative properties in experimental arthritis^2^. Similarly, in the present study we show that the pharmacologically-induced glycolytic switch of MSC PPARβ/δ^+/+^ significantly enhanced their immunoregulatory potential to an even greater extent than MSC PPARβ/δ^−/−^. Thus, these results suggest that the immunoregulatory potential of MSC involves other metabolic pathways than those related to PPARβ/δ. Indeed, recently it has been demonstrated that mitochondrial transfer from MSC to T cells represents a novel mechanism of immunosuppression involved in the inhibition of Th17 cell proliferation and function as well as in the generation of regulatory T cells to restrain inflammation^13,14^. Thus, the improved immunosuppressive activity of MSC PPARβ/δ^−/−^ could be associated to their higher mitochondrial transfer capacity as compared to MSC PPARβ/δ^+/+^. This hypothesis provides the basis for further investigations.

The enhancement of MSC immunoregulatory activity either using pro-inflammatory cytokines or inhibiting PPARβ/δ expression is associated with an increase glycolytic activity of MSC. This is in line with a recent study showing that IFNγ and hypoxia double priming of MSC enhances twice more their immunosuppressive properties than a single priming through a glycolytic switch of dual-primed MSC^15^. Therefore, this metabolic switch of MSC towards glycolysis leading to lactate production and the inhibition of T cells proliferation might be the key that permits, extends and guarantees MSC therapeutic effects regardless the window of injection.

These findings highlight the importance of such metabolic reprogramming for MSC immunoregulatory potential and pave the way for an enhanced MSC-based therapy for inflammatory and auto-immune disorders.

## Material and methods

### Bioethics

All methods were carried out in accordance with relevant guidelines and regulations for using animals. All the procedures presented in this work were approved by the Ethics Committee of Universidad de los Andes (Folio CEC Nº201630, Universidad de los Andes, Santiago, Chile).

### Isolation and culture of MSC

Murine MSC were obtained from bone marrow of 129/Sv PPARβ/δ-deficient mice (*Ppard*^*fl/fl*^ sox2cre^tg^) referred as PPARβ/δ^−/−^ MSC and their wild-type littermates (*Ppard*^*fl/*+^) referred as PPARβ/δ^+/+^ MSC. Murine MSCs were characterized as previously described^16^. Murine MSC were cultured in Dulbecco’s modified eagle medium (DMEM) high glucose (Corning, USA), and supplemented with 10% Fetal Bovine Serum, 1% Pen/Strep and 1% glutamine (Gibco, Thermo Fisher, USA). All the procedures presented in this work were approved by the Ethics Committee of Universidad de los Andes.

### Immunosuppression assay

CD4^+^ T cells were freshly isolated from spleen of C57BL/6 mice by negative selection with Dynabeads Untouched Mouse CD4 Cells Kit (Invitrogen, Thermo Fisher, USA) according to manufacturer’s instructions. Once purified, they were labeled with CellTrace Violet (CTV) (Life-Technology, Thermo Fisher, USA) and activated with CD3/CD28 beads (Invitrogen, Thermo Fisher, USA). Lymphocytes were cultured in mixed lymphocyte reaction (MLR) media, containing 10% Fetal Bovine Serum, 1% Pen/Strep, 1% sodium pyruvate, 1% non-essential amino acids, 1% glutamine and 25 µM β-mercaptoethanol (Gibco, Thermo Fisher, USA), in Iscove's Modified Dulbecco's Media (IMDM) (Gibco, Thermo Fisher, USA). To differentiate towards Th1 subtype, purified CD4^+^ T cells were stimulated with 10 ng/ml of IL12 (R&D Systems, USA) and 2.5 μg/ml of anti-IL4 (BD Pharmingen, BD Biosciences, USA). Similarly, Th17 phenotype was induced with 50 ng/ml of IL6 (R&D Systems, USA), 2.5 ng/ml of TGBβ1 (R&D Systems, USA), 2.5 μg/ml of anti-IFNγ (BD Pharmingen, USA) and 2.5 μg/ml of anti-IL4 (BD Pharmingen, USA).

To assess immunosuppressive properties of murine MSCs, CD4^+^ T cells were cultured alone or in the presence of MSCs (control vs pretreated) at a cell ratio of 1 MSC per 10 lymphocytes in MLR media. After 72 h, proliferation and CD4^+^ T cell differentiation was quantified by flow cytometry.

### Flow cytometry

Proliferation and differentiation of lymphocytes were quantified by flow cytometry. T cells were stimulated with phorbolmyristate acetate (PMA) (50 ng/ml; Merck, Germany) and ionomycin (1 mg/ml; Merck, Germany), in the presence of brefeldin A (10 mg/ml; Sigma, Merck, Germany) for 4 h. Then surface staining was performed together with LIVE/DEAD Fixable near-IR stain (Invitrogen, Thermo Fisher, USA) in order to achieve the analysis only in live cells. Then, cells were fixed at 4 °C with the FoxP3 Cytofix/Cytoperm buffer (eBioscience, USA) and subsequently stained with intracellular fluorochrome-conjugated antibodies diluted in Perm/Wash buffer (eBioscience, USA) according to manufacturer’s specifications.

### Seahorse assay

Using the XF96 analyzer (Seahorse Biosciences, North Billerica, MA, USA), we measured the Oxygen Consumption Rate (OCR) and Extracellular Acidification Rate (ECAR), associated to oxidative phosphorylation and secretion of lactic acid as a metabolic product of glycolysis, respectively. Pre-stimulated murine MSC (20.000 cells/well) were plated on 96 well plates and analyzed according to manufacturer’s recommended protocol. Three independent readings were performed after each sequential injection. Instrumental background was measured in separate control wells using the same conditions without biologic material.

Basal glycolytic rate was measured following the injection of glucose injection. Maximal glycolytic level was assessed following the injection of oligomycin and glycolytic capacity as the difference of oligomycin-induced ECAR and 2DG-induced ECAR. In XF media, OCR has been quantified under different conditions including basal conditions, in response to 1 μM oligomycin, 1 μM of FCCP (carbonylcyanide-4-(trifluoromethoxy)-phenylhydrazone) or 1 μM of antimycin A and rotenone (Sigma Aldrich).

### Reverse transcription-polymerase chain reaction (PCR) and quantitative real-time PCR (qRT-PCR)

We performed reverse transcription PCR and qRT-PCR as we previously described^17^. Gene expression relative changes were calculated by the relative quantification method (2^−ΔCt^).

### Quantification of immunosuppressive molecules produced by MSC PPARβ/δ^+/+^ and MSC PPARβ/δ^−/−^

In order to study the expression levels of the immunosuppressive molecules IL6 and TGFβ1 was quantified by ELISA Kit (R&D System, FR) according to manufacturer’s instructions and the protein expression levels of PD-L1, VCAM and ICAM (BD biosystem, USA), and GLUT1 (Metafora, FR) were measured by FACS. Nitric oxide was quantified using a modified griess reaction as we previously described^12^.

### Metabolites quantification

Glucose and lactate concentrations were measured using an Analyzer Y15 (BioSystems S.A., Spain).

### Statistical analysis

Results were expressed as the mean ± SD. All in vitro experiments were performed using four different biological replicates at least four independent times. The p values were generated using non-parametric analysis using the Mann–Whitney *U* test to compare between two groups; p < 0.05 (*), p < 0.01 (**) or p < 0.001 (***) were considered statistically significant. All the analyses were performed using the GraphPad Prism 6 software (Graphpad Software, San Diego, California, USA).

## Supplementary information

Supplementary information
