# Supplementary material for: PPARβ/δ-dependent MSC metabolism determines their immunoregulatory properties
Source: Sci Rep. 2020 Jul 10;10:11423. doi: 10.1038/s41598-020-68347-x (PMC7351754; doi:10.1038/s41598-020-68347-x)
Supplement: Supplementary file 1 — Supplementary information [file 41598_2020_68347_MOESM1_ESM.pdf]

# PPARß/δ-dependent MSC metabolism determines their immunoregulatory properties

*Authors:*

Contreras-Lopez RA[1,6#], Elizondo-Vega R[4#], Torres MJ[7], Vega-Letter AM[1], Luque-Campos N[1,6,], Paredes-Martinez MJ[1], Pradenas C[1], Tejedor G[3], Oyarce K[5], Salgado M[4], Jorgensen C[3], Khoury M[2], Kronke G[8], Garcia-Robles MA[4], Altamirano C[7], Luz-Crawford P[1]* and Djouad F[3]*

*Affiliations:*

[1]Centro de Investigación Biomédica, Facultad de Medicina, Universidad de los Andes, Santiago, Chile; [2]Cells for Cells, Universidad de los Andes, Santiago, Chile; [3]IRMB, Univ Montpellier, INSERM, Montpellier, France; [4]Facultad de Ciencias Biológicas, Departamento de Biología Celular, Laboratorio de Biología Celular, Concepción, Chile; [5]Facultad de Ciencias de la Salud, Universidad San Sebastián, Concepción, Chile.

; [6]Facultad de Medicina, Universidad de los Andes, Santiago, Chile; [7]Escuela de Ingeniería Bioquímica, Valparaiso, Chile; [8]Department of Internal Medicine 3, University of Erlangen-Nuremberg, Erlangen 91054, Germany;

# and * Equally contributing author

***Keywords:*** *Mesenchymal Stem Cell, Metabolism, reprogramming, glycolytic, immunoregulation*

*Correspondence to:*

Farida Djouad, Inserm U 1183, IRMB, Hôpital Saint-Eloi, 80 avenue Augustin Fliche, 34295 Montpellier cedex 5, France

Tel: 33 (0) 4 67 33 04 75 – E-mail: farida.djouad@inserm.fr

Patricia Luz-Crawford, Centro de Investigación Biomédica, Facultad de Medicina, Universidad de los Andes, Santiago, Chile.

Tel: 56 (2) 26181481 – E-mail: pluz@uandes.cl

## Supplementary 1

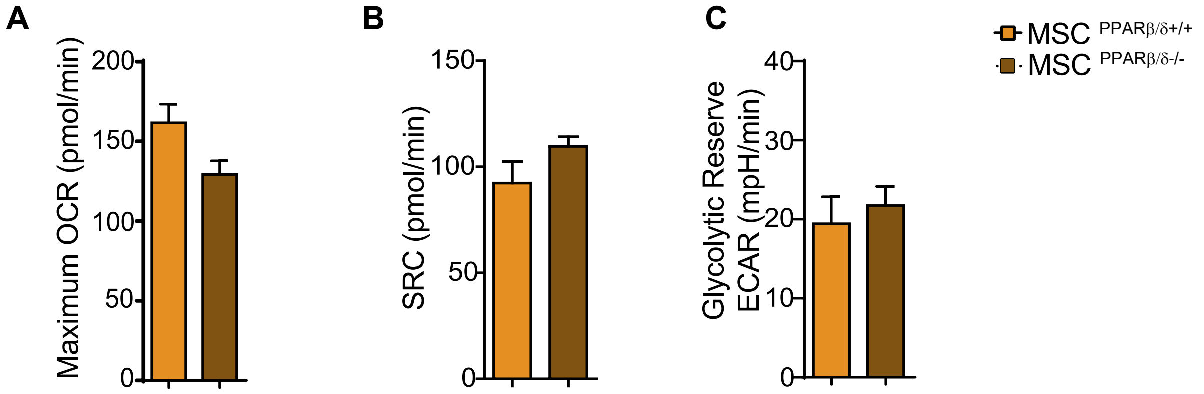

**Supplementary Figure 1. PPARβ/δ expression induces an OXPHOS dependent metabolism on murine MSC. (A-C)** The metabolic status of PPARβ/δ$^{+/+}$ or PPARβ/δ$^{-/-}$ MSC was determined by analyzing the maximum oxygen consumption rates (OCR) **(A)**, the spare respiratory capacity (SRC) **(B)** and the glycolytic reserve **(C)** using the Agilent Seahorse XF technology**.** Results are represented as mean ± SD of at least 4 independent experiments.

# Supplementary 2

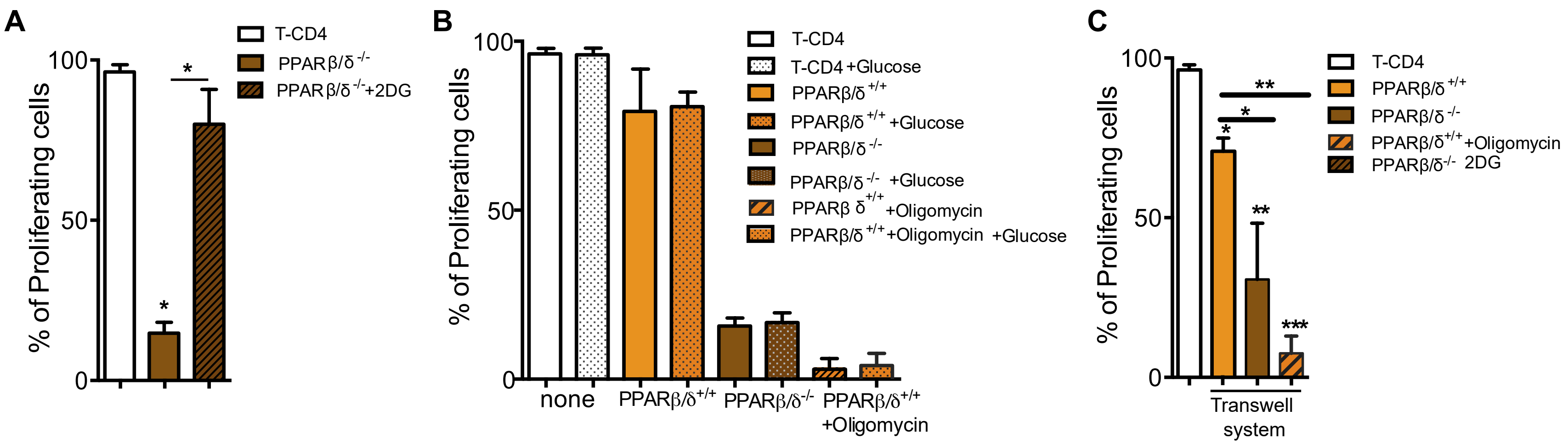

**Supplementary Figure 2. Glucose addition in the culture media do not modify MSC immunosuppressive properties. (A)** Naïve T-CD4 murine cells labelled with Cell Trace Violet (CTV) were cultured with MSC PPARβ/δ$^{-/-}$ pre-treated (brown bars) or not (lined brown bars) with 2DG. **(B)** Naïve T-CD4 murine cells were labelled with Cell Trace Violet (CTV) prior to be activated. Then, the cells were cultured alone (white bars) or with either MSC PPARβ/δ$^{+/+}$ pre-treated (lined yellow bars) or not (yellow bars) with oligomycin or MSC PPARβ/δ$^{-/-}$ (brown bars). Glucose was added when indicated in the culture media at 25mM (white and colored bars with dot). **(C)** Naïve T-CD4 murine cells labelled with Cell Trace Violet (CTV) were cultured alone (white bars) or with MSC PPARβ/δ$^{+/+}$ pre-treated (lined yellow bars) or not (yellow bars) with oligomycin or MSC PPARβ/δ$^{-/-}$ (brown bars) using a transwell system. Proliferation, was evaluated by FACS. Statistics: non-paired Kruskal-Wallis test.
